# *Aethesoma prosternistriatum*, a new genus and species from southern China (Coleoptera, Histeridae, Histerinae, Platysomatini)

**DOI:** 10.3897/zookeys.1295.208722

**Published:** 2026-10-23

**Authors:** Jihuan Zheng, Tomáš Lackner

**Affiliations:** 1 Guangdong Key Laboratory of Animal Conservation and Resource Utilization, Institute of Zoology, Guangdong Academy of Sciences, Guangzhou, China Guangdong Key Laboratory of Animal Conservation and Resource Utilization, Institute of Zoology, Guangdong Academy of Sciences Guangzhou China; 2 Naturhistorisches Museum Basel, Augustinergasse 2, 4051 Basel, Switzerland Naturhistorisches Museum Basel Basel Switzerland

**Keywords:** Guangdong, Guangxi, identification key, Jiangxi, morphological comparison, taxonomy

## Abstract

A new histerid genus *Aethesoma***gen. nov**., with a new species *Aethesoma prosternistriatum***gen. et sp. nov**. (Histeridae, Histerinae, Platysomatini) is described. The new species is most likely a subcortical dendrobiont distributed in Guangdong, Guangxi, and Jiangxi provinces of southern China and was collected by flight-interception traps. *Aethesoma* is morphologically distinct from all other known genera of Platysomatini. A key to Chinese Platysomatini is provided.

## Introduction

Platysomatini, established by Bickhardt (1914), is a tribe of clown beetles (Coleoptera: Histeridae), a diverse family characterized by considerable morphological and ecological diversity (Mazur and Ôhara 2000a). The tribe currently comprises 30 genera (Mazur 2011) and approximately 380 described species. The most species-rich genera are *Platylister* Lewis, 1892 and *Platysoma* Leach, 1817, whereas other well-represented genera include *Apobletes* Marseul, 1861, *Eblisia* Lewis, 1889, and *Eurylister* Bickhardt, 1920. In China, 53 species belonging to 14 genera have been recorded to date, although the actual diversity is likely to be considerably higher (Zhou et al. 2022).

Members of Platysomatini are distinguished by a combination of morphological characters, including: (i) V-shaped antennal club sutures; (ii) an S-shaped protarsal groove; (iii) a moderately sclerotized, dorsoventrally flattened median lobe of the aedeagus bearing a slender basal process; and (iv) a straight lateral metaventral stria (Bickhardt 1917; Kryzhanovskij 1972; Ôhara 1994). The taxonomy and phylogenetic relationships of the tribe have been the subject of several comprehensive studies (Mazur and Ôhara 2000a, 2000b, 2009; Ôhara and Mazur 2000, 2002). These studies demonstrated that Platysomatini, as circumscribed by Mazur (1997), are not monophyletic and comprise several distinct evolutionary lineages warranting recognition as separate taxonomic groups. Morphological characters such as the configuration of the protarsal groove, frontal striae, and male genitalia have proven particularly informative for delimiting genera and species within the tribe.

Herein, we describe a new genus and species of Platysomatini from southern China. In addition, we provide an identification key to all genera of Platysomatini currently known from China.

## Materials and methods

Specimens were collected from Dinghushan (mountain) Natural Reserve, Guangdong Province (Fig. 4), Huaping National Nature Reserve, Guangxi Zhuang Autonomous Region and Jiulianshan Natural Reserve, Jiangxi Province, southern China in 2020–2021 by flight-interception traps. Morphological characters were examined with Zeiss stemi305 microscope. Male genitalia were dissected according to Zheng and Lackner (2025).

Morphological concepts and body measurements used in this study mostly follow Ôhara (1994); leg terminology follows Lackner (2010). Abbreviations for measurements of body parts are as follows: **PEL** = length between anterior angles of pronotum and apices of elytra; **PL** = length of pronotum along midline; **APW** = width between anterior angles of pronotum; **PPW** = width between posterior angles of pronotum; **EL** = length of elytron along sutural line; **EW** = maximal width between outer margins of elytra; **PgL** = length of pygidium along midline; **PgW** = maximal width between outer margins of pygidium.

Habitus images were taken using a Sony a7 digital camera. Aedeagal images were taken using Nikon D610 digital camera, attached to a Zeiss V/A1 microscope with 5× objective lens. All images were stacked using Helicon Focus v. 7 to obtain the full depth of focus, and the resulting output was edited with Adobe Photoshop 2025.

Specimens are deposited at the Institute of Zoology, Guangdong Academy of Sciences, Guangzhou, China (**GIZ**), as well as authors’ private collections (Jihuan Zheng, Guangzhou, China – **CJZH**; Tomáš Lackner, Munich, Germany – **CTLA**).

## Taxonomy

**Family Histeridae Gyllenhal, 1808**

**Subfamily Histerinae Gyllenhal, 1808**

**Tribe Platysomatini Bickhardt, 1914**

### Aethesoma

Taxon classificationAnimaliaColeopteraHisteridae

gen. nov .

FEB85128-E01C-521A-A230-E2434F548315

https://zoobank.org/E9B73E1F-C82A-4E39-A90A-27A7E339FAA0

#### Type species.

*Aethesoma prosternistriatum* sp. nov.

#### Diagnosis.

Medium-sized dark-brown-to-black Platysomatini beetle with elongate-oval body; head with frontal stria and disk concave; pronotum punctate; elytra completely striate; pygidium with raised margin and covered with large punctures; prosternum with carinal striae; metaventrite laterally bistriate; first visible abdominal sternite bistriate laterally.

#### Differential diagnosis.

*Aethesoma* gen. nov. can be recognized from other genera of Platysomatini by the following combination of characters: pronotum with variable-sized punctures; prosternal keel moderately broad; marginal prosternal stria present; prosternal process with two sets of prosternal striae; meso-metaventral stria absent; lateral metaventral stria doubled; propygidium and pygidium deeply punctate.

*Aethesoma* gen. nov. exhibits a raised pygidial margin similar to that of the genus *Platylister* (s. str.) Lewis, 1892. Punctation on the pronotum is comparable to that of Platysoma (Cylister) Cooman, 1941. The meso-metaventral marginal and lateral striation are similar to that found in the genus *Nicotikis* Marseul, 1883.

*Aethesoma* gen. nov. is morphologically closest to the genus *Platysoma* Leach, 1817, sharing the following characters: disk of head depressed, anterior margin of labrum medially emarginate, pronotum with variable-sized punctures, dorsal elytral striation present, prosternal keel moderately broad, lateral metaventral stria double. *Platysoma* is immediately distinguishable from *Aethesoma* in having the prosternal keel without carinal prosternal striae and the pygidium usually not marginally elevated. *Platysoma* is divided into three subgenera based on the degree of body elongation (the length-to-width ratio of the mesoventrite), *Platysoma* s. str., *Cylister* Cooman, 1941 and *Cylistus* Dejean, 1833, but none of these subgenera exhibit mesoventral and pygidial characters that depart from the genus.

*Niposoma* Mazur, 1999 shares its general shape and some characteristics of the pronotum and legs with *Aethesoma* gen. nov., such as outer edge of lateral pronotal stria being strongly carinate laterally and coarse punctures present laterally. *Niposoma* specifically differs from *Aethesoma* gen. nov. in having a single lateral metaventral stria.

*Aethesoma* gen. nov. exhibits certain similarities with *Nicotikis* in its meso-metaventral marginal and lateral striation, but it differs from *Nicotikis* in possessing an outer lateral stria on the pronotum and punctation on its disc, in the absence of a pygidial fovea, and in the shape of the prosternal process (narrow in *Nicotikis*) (Mazur 2007; Zinchenko 2014).

Regarding the comparative discussion on male genital morphology of the Chinese species (e.g. Zhou et al. 2022), there are several similarities among taxa, yet these preclude definitive statements on the usefulness of these characters. For example, Platylister (Popinus) dahdah (Marseul, 1861) exhibits a Y-shaped ninth sternite (spiculum gastrale), while others, for example Platylister (Platylister) cambodjensis (Marseul, 1864) and Platylister (Platylister) suturalis (Lewis, 1888) possess an I-shaped one, similar to that of *Aethesoma* gen. nov. As mentioned below, the tribe is in need of thorough phylogenetic examination.

#### Etymology.

*Aethesoma* is a neuter noun derived from the Ancient Greek words aḗthēs (ἀήθης), meaning “unusual,” “strange,” “unfamiliar,” or “unaccustomed,” and sôma (σῶμα), meaning “body.” The generic name refers to the unusual body morphology of the new taxon, which cannot be assigned to any previously known genus based on its morphological characters. Thus, the name emphasizes the distinctive and atypical habitus of the genus.

### Aethesoma prosternistriatum gen. et sp. nov.

Taxon classificationAnimaliaColeopteraHisteridae

9626BE70-88DE-5F7A-B270-4F2E236D6B44

https://zoobank.org/2A6D3C47-4742-4B51-B8F9-477387164DD3

Figs 1, 2, 3

#### Type material.

***Holotype***: ♂, with the following labels: 广东鼎湖山保护区 [= CHINA, Guangdong, Zhaoqing, Dinghushan Nature Reserve], 23°10'18.01"N, 112°32'34.36"E, 2021.VIII.4–VIII.11, FIT-7 [= flight-interception trap], 采集人：杨海东 [= Haidong Yang leg]. // HOLOTYPE [red label, printed] (GIZ).

***Paratypes***: 1 ♂, 江西龙南九连山 [= CHINA, Jiangxi, Longnan, Jiulianshan Natural Park] 24°32'15.58"N, 114°27'52.80"E, 2021.VI.5–10, FIT, 采集人：杨海东 [= Haidong Yang leg] // PARATYPE [yellow label, printed] (GIZ). 1 ♀, 江西龙南九连山 [= CHINA, Jiangxi, Jiulianshan, Nature Reserve], 24°35'14.58"N, 114°27'6.77"E, 2021.V.21–V.25, FIT-8, 采集人：杨海东 [= Haidong Yang leg]. // PARATYPE [yellow label, printed] (GIZ). 2 ♀, 广东鼎湖山保护区[= CHINA, Guangdong, Zhaoqing, Dinghushan Nature Reserve], 23°10'3.14"N, 112°32'46.60"E, 2021.V.25–28, FIT-10, 采集人：杨海东 [= Haidong Yang leg]. // PARATYPE [yellow label, printed] (GIZ). 1 unsexed specimen, 广东鼎湖山保护区 [= CHINA, Guangdong, Zhaoqing, Dinghushan Nature Reserve], 23°9'27.93"N, 112°32'45.46"E, 2021.V.31–VI.3, PIT-3, 采集人：杨海东 [= Haidong Yang leg]. // PARATYPE [yellow label, printed] (CTLA). 1 unsexed specimen, 广东鼎湖山保护区 [= CHINA, Guangdong, Zhaoqing, Dinghushan Nature Reserve], 23°9'33.96"N, 112°32'58.50"E, 2021.VI.6–VI.9, 采集人：杨海东 [= Haidong Yang leg]. // PARATYPE [yellow label, printed] (CJZH). 1 unsexed specimen, 广西花坪 [= CHINA, Guangxi, Guilin, Huapingshan Nature Reserve], 2020. V. 24, FIT, 采集人：杨海东 [= Haidong Yang leg]. // PARATYPE [yellow label, printed] (GIZ).

#### Description.

**Figure 1.**
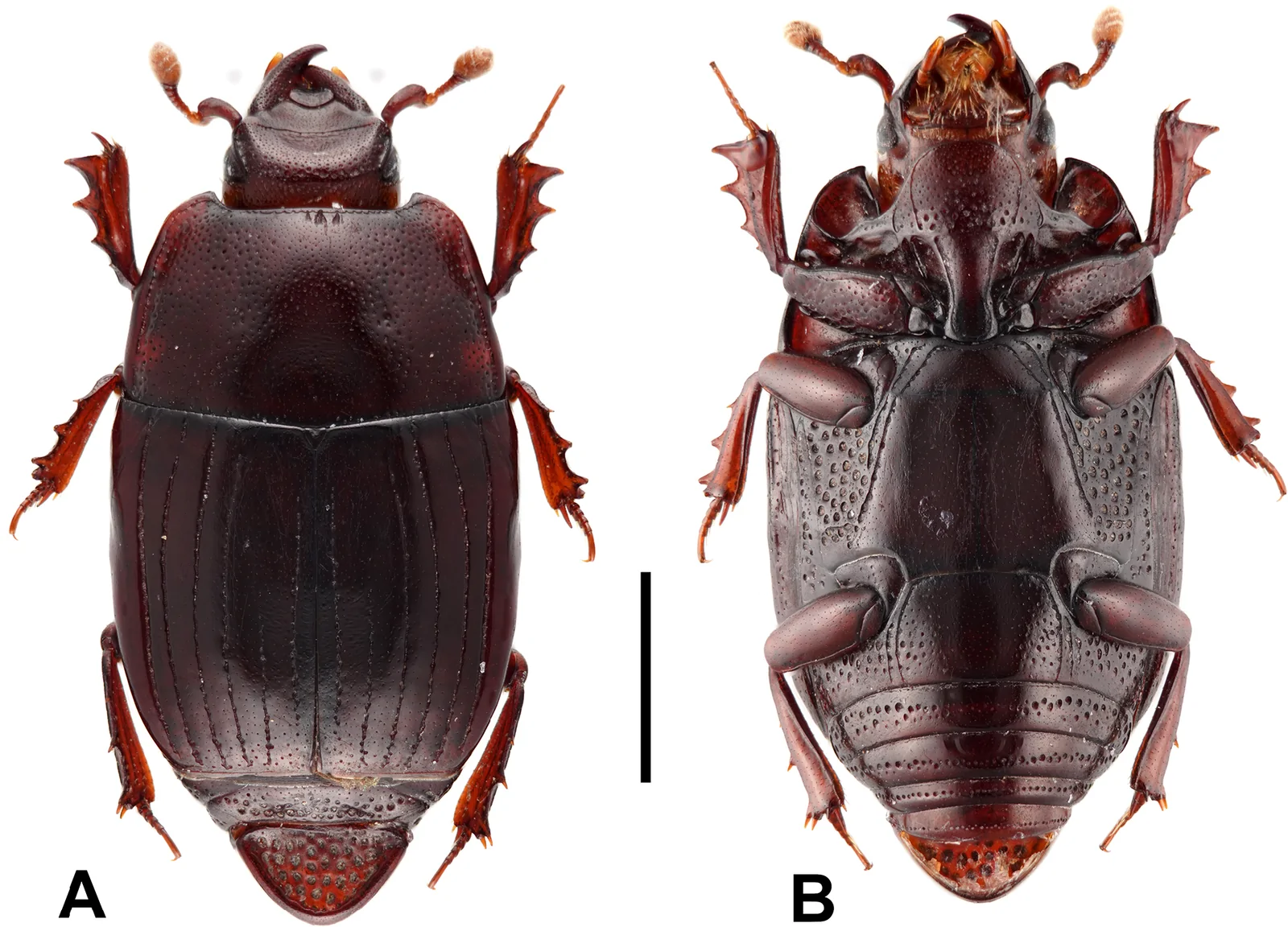
Habitus of *Aethesoma prosternistriatum* gen. et sp. nov. **A**. Dorsal view; **B**. Ventral view. Scale bar: 1 mm.

***Body***: length: 2.85–3.19 mm (PEL, average = 2.99 mm, *n* = 8); width: 1.79–1.99 (EW, average = 1.90 mm, *n* = 8). Full biometric data are given in Table 1. Body elongate, reddish brown, shining; antennae, tibiae and tarsi paler (Fig. 1A, B).

**Table 1.** Biometric data of *Aethesoma prosternistriatum* sp. nov. Abbreviations for measurements of body parts follow Ôhara (1994) and are explained in Materials and methods.

**Specimen**	**PEL**	**PL**	**APW**	**PPW**	**EL**	**EW**	**PgL**	**PgW**
1	2.96	1.16	0.99	1.76	1.94	1.79	0.53	0.76
2	2.85	0.99	0.93	1.71	1.69	1.87	0.45	0.68
3	2.93	1.20	0.98	1.83	1.85	1.91	0.48	0.75
4	2.99	1.23	1.06	1.82	1.86	1.88	0.54	0.71
5	2.91	1.04	1.05	1.79	1.79	1.91	0.46	0.76
6	3.19	1.09	1.03	1.86	1.86	1.99	0.59	1.02
7	3.03	1.04	0.98	1.75	1.95	1.92	0.55	0.88
8	3.02	1.14	1.04	1.80	1.95	1.95	0.56	0.90
Average	2.99	1.11	1.01	1.79	1.86	1.90	0.52	0.81

***Head***: frontal stria complete and well impressed; disk deeply concave, sparsely and finely punctate.

***Pronotum***: pronotal disk feebly convex medially; marginal pronotal stria complete laterally, anteriorly absent. Lateral pronotal stria deeply impressed, complete. On apical third this stria feebly sinuate, medially strongly carinate, becoming weakly carinate on both ends. Surface of pronotum densely covered with coarse, deep punctures growing in size laterally. Punctures intermingled with microscopic punctation.

***Elytra***: epipleural disk slightly excavate, with fine punctures. Elytral epipleuron with two marginal epipleural striae: outer stria finely impressed and broadly interrupted on median third; inner stria clearly impressed, complete, carinate. Marginal elytral stria deeply impressed, complete, its outer margin strongly carinate. Subhumeral stria absent. Oblique humeral stria present on basal third. First to fourth dorsal elytral striae complete and deeply impressed with sparse, coarse punctures; fifth dorsal elytral stria present on apical two-thirds; short rudiment basally present. Sutural elytral stria almost complete, basally and apically slightly abbreviated, sinuate on apical half. Elytral disk feebly convex medially; surface with microscopic punctures.

**Figure 2.**
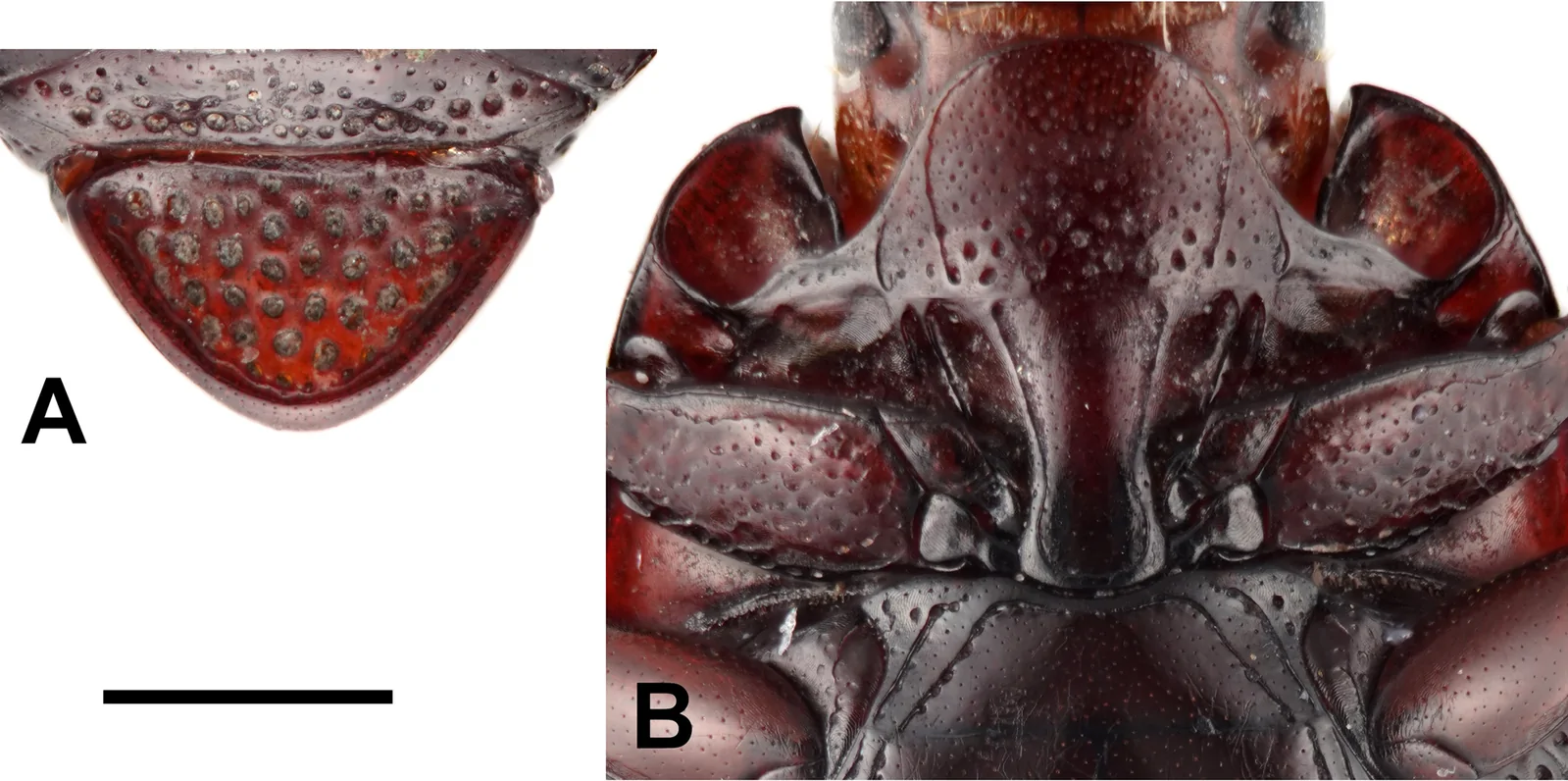
Diagnostic features of *Aethesoma prosternistriatum* gen. et sp. nov. **A**. Propygidium and pygidium; **B**. Prosternal lobe and process. Scale bar: 0.5 mm.

***Pygidia***: propygidium (Fig. 2A) transverse, covered with variously sized punctures, becoming distinctly large and deep along posterior margin. Pygidium subtriangular, sparsely covered with large, deep, round punctures, their inter-distances shorter than their diameter; margin of pygidium strongly elevated.

***Prosternum***: prosternal lobe (Fig. 2B) broad, anteriorly rounded; margined by a well-impressed stria that is widely interrupted antero-medially. A complementary short stria present basally. Disk of prosternal lobe with fine deep punctures growing in size and depth bassally. Prosternal process moderately broad, finely punctate. Carinal prosternal striae deeply impressed, apically divergent, roundly united on prosternal apophysis. Lateral prosternal striae doubled, costate, apically widely divergent.

***Meso-metaventrite***: anterior margin of mesoventrite medially emarginate; marginal mesoventral stria complete and subcariniform; a complimentary stria present, running parallel to it, widely interrupted at middle. Mesoventral disk with sparse microscopic punctures. Meso-metaventral suture visible; stria absent. Metaventrite likewise laterally bistriate; inner lateral stria posteriorly extending, stopping short of metacoxa; outer lateral stria apically abbreviated. Lateral disk of metaventrite densely covered with large, round, deep punctures intermingled with microscopic punctuation.

***Abdomen***: intercoxal disk of first visible abdominal sternite very sparsely clothed with microscopic punctures, with several large punctures laterally. Laterally disk completely bistriate.

***Legs***: protibia (Fig. 1) with three wide, triangular teeth diminishing in size towards proximal end; each tooth topped by a tiny denticle. Fourth, proximal-most tooth microscopic. Protibial spur conspicuous. Mesocoxa with a distinct cariniform stria. Mesotibia slightly dilated, with three widely separated triangular teeth topped by a denticle; apical-most tooth double. Mesotibial spur strongly developed. Metatibia more slender, with a single medial denticle; near tarsal insertion with a doubled tooth topped by conspicuous denticle.

**Figure 3.**
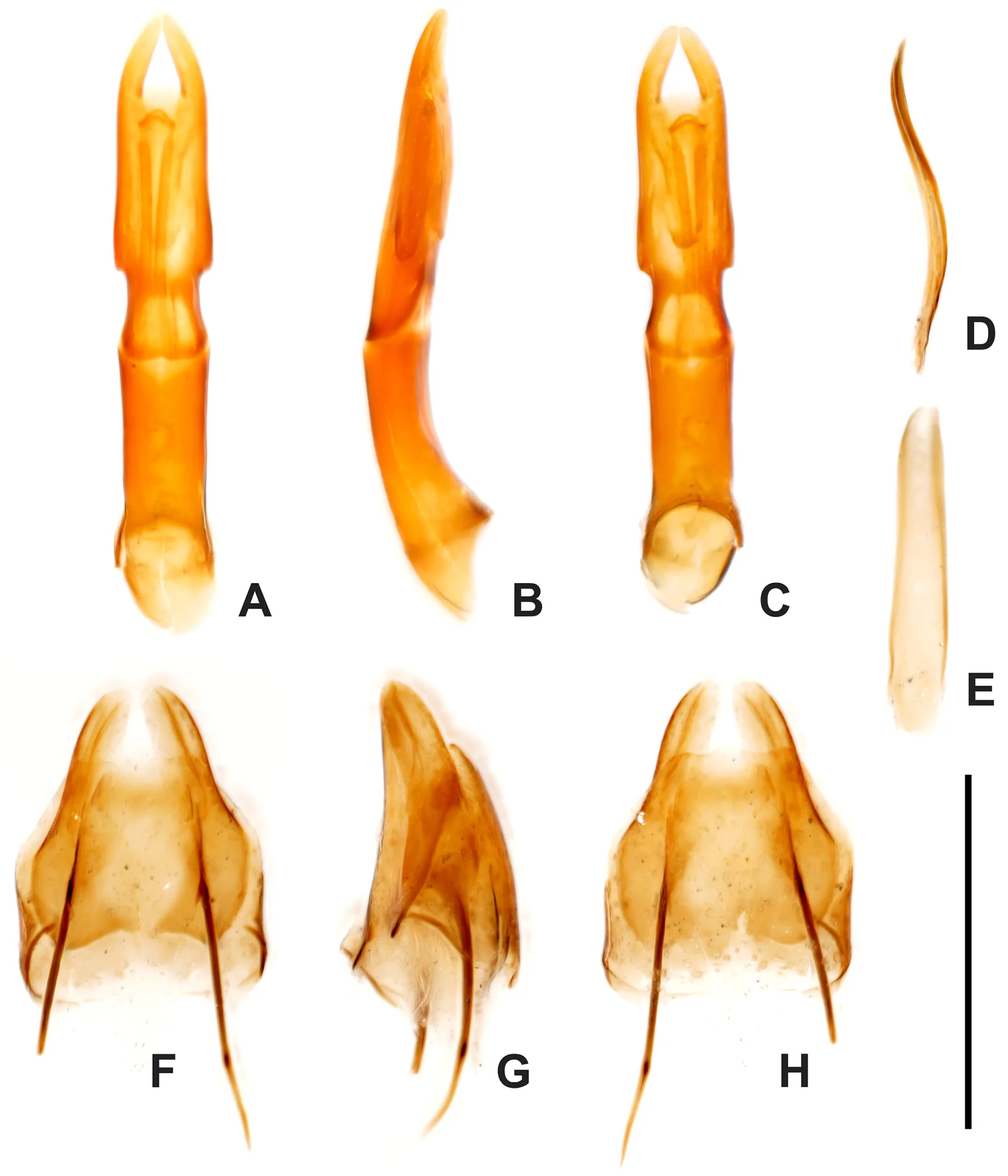
Male terminalia of *Aethesoma prosternistriatum* gen. et sp. nov. **A**. Aedeagus, dorsal view; **B**. Ditto, lateral view; **C**. Ditto, ventral view; **D**. Ninth sternite (spiculum gastrale), lateral view; **E**. Ditto, dorsal view; **F**. Eighth sternite and tergite+ninth and tenth tergites, dorsal view; **G**. Ditto, lateral view; **H**. Ditto, ventral view. Scale bar: 0.5 mm.

***Male genitalia***: eighth sternite and tergite apically narrowed; ninth tergite with long processes (tails) at base. Ninth sternite (spiculum gastrale) I-shaped, bisinuate in lateral view. Aedeagus: ratio in length of parameres to basal piece of aedeagus 1.1, base of tegmen constricted, thence running subparallel; parameres apically open; apex acute (Fig. 3).

#### Etymology.

The specific epithet *prosternistriatum* refers to the prominent striation of the prosternal process, which represents one of the diagnostic characters of the species.

#### Distribution.

**Figure 4.**
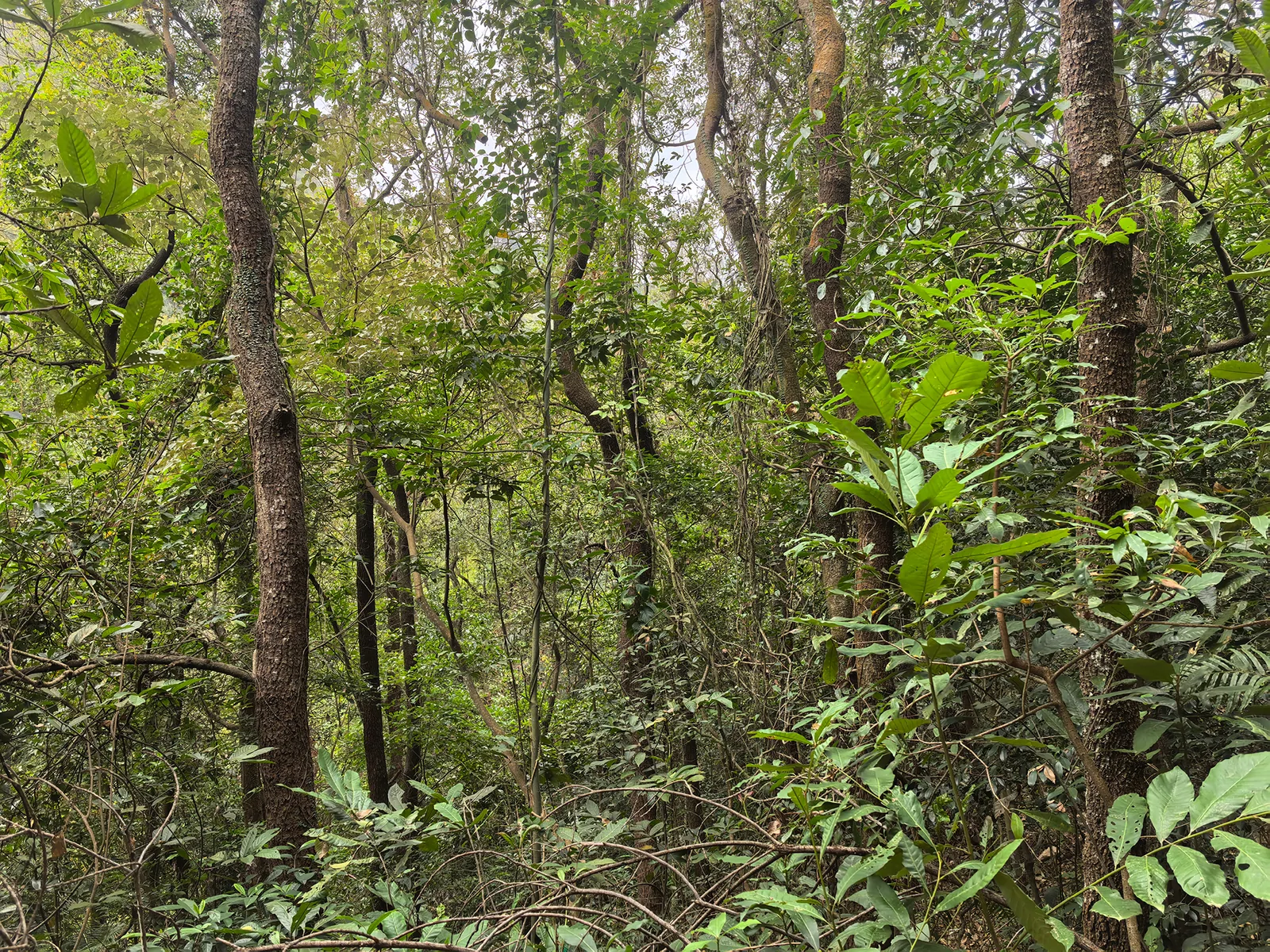
Type locality of *Aethesoma prosternistriatum* gen. et sp. nov.: Dinghushan (mountain), Guangdong Province, southern China (Photo by Zongji Fan).

Southern China (Guangdong, Guangxi, Jiangxi). The type locality, Dinghushan in Guangdong Province (Fig. 4) is a lowland forest at approximately 200 m elevation and features a subtropical monsoon climate characterized by relatively long hours of sunshine and abundant solar radiation. There are no frigid winters or scorching summers, and the region remains warm year round.

#### Remarks.

The new species appears to be rare; only eight specimens have been collected from three localities. Phenotypic variation and sexual dimorphism have not been observed in available specimens collected from three provinces.

##### Key to genera of Chinese Platysomatini

Modified from Mazur and Ôhara 2009; Zhou et al. 2022.

**Table:** 

1	Mandibles stout; inner margin with a subapical tooth	**2**
–	Mandibles elongate; apex bifid; subapical tooth absent	***Megagnathos* Penati & Zhang, 2009**
2	Protarsal groove usually S-shaped, long and deep	**3**
–	Protarsal groove usually straight, short and shallow	***Mendelius* Lewis, 1908**
3	Anterior margin of labrum arcuate	**4**
–	Anterior margin of labrum emarginate or straight	**5**
4	Mandibles without groove; pygidium with two large and deep excavations near the outer edge	***Liopygus* Lewis, 1891**
–	Base of mandibles with groove; pygidium without excavations	***Silinus* Lewis, 1907**
5	Body large (>9.00 mm), protibia with two large, blunt teeth	***Plaesius* Erichson, 1834**
–	Body small to medium-sized (<9.00 mm), protibia with at least three short teeth	**6**
6	Prosternum with one lateral stria; external subhumeral stria complete	***Aulacosternus* Marseul, 1853**
–	Prosternum with two lateral striae; external subhumeral stria vestigial or outright absent	**7**
7	Prosternal process very broad; body extremely flat	***Apobletes* Marseul, 1861**
–	Prosternal process moderately broad or narrow; body more convex	**8**
8	Mesocoxa without a cariniform stria	**9**
–	Mesocoxa with a distinct cariniform stria	**10**
9	Lateral pronotal stria approximate to pronotal margin; inner lateral metaventral stria bisinuate, outer one shortened	***Eurylister* Bickhardt, 1920**
–	Lateral pronotal stria distanced from pronotal margin; inner lateral metaventral stria usually straight, outer one extending posteriorly, approximately as long as inner one apically	***Eblisia* Lewis, 1889**
10	Pronotum laterally without coarse punctures	***Platylister* Lewis, 1892**
–	Pronotum laterally with coarse punctures	**11**
11	Metaventrite with a single lateral metaventral stria	**12**
–	Metaventrite with two subparallel lateral metaventral striae	**14**
12	Prosternum without carinal prosternal striae	***Kanaarister* Mazur, 1999**
–	Prosternum with carinal prosternal striae	**13**
13	Body elongate (PEL/EW > 1.50 mm); lateral pronotal stria distanced from pronotal margin, sinuous	***Niposoma* Mazur, 1999**
–	Body oval (PEL/EW < 1.50 mm), lateral pronotal stria parallel to pronotal margin	***Eurosomides* Newton, 2015**
14	Prosternum with carinal prosternal striae	***Aethesoma* gen. nov**.
–	Prosternum without carinal prosternal striae	***Platysoma* Leach, 1817**

## Discussion

Mazur and Ôhara (2000a) highlighted the taxonomic complexity of the tribe Platysomatini and outlined an ambitious program to redescribe all recognized genera and subgenera while critically reassessing the tribe’s taxonomy. Their ultimate objective was to establish a revised classification based on a comprehensive morphological phylogenetic analysis. Although this initiative resulted in a series of detailed, predominantly descriptive taxonomic studies (see references above), the anticipated comprehensive phylogenetic treatment was never completed. Likewise, a diagnostic key to the higher taxa of Platysomatini has yet to be published. Consequently, the taxonomy of the tribe remains unstable, rendering the identification of specimens and their assignment to higher taxa difficult and often uncertain.

In the absence of a comprehensive identification key, we relied primarily on the taxonomic revisions of Mazur and Ôhara (see Introduction for references), supplemented by the recently published treatment of the Chinese Histeridae fauna (Zhou et al. 2022). Despite these efforts, we were unable to assign our material to any currently recognized genus. We discussed the matter with Prof. Mazur, Prof. Ôhara, and several other specialists in histerid taxonomy; however, none could confidently place these specimens within an existing genus.

Although we are reluctant to further complicate the already unsettled taxonomy of Platysomatini, we concluded that the erection of a new genus, *Aethesoma* gen. nov., is the most appropriate taxonomic solution at present. The unique combination of morphological characters exhibited by these specimens precludes their confident placement in any currently recognized genus. We acknowledge that future phylogenetic studies integrating both molecular and morphological data may demonstrate that *Aethesoma* gen. nov. is synonymous with an existing taxon. Nevertheless, given the current state of knowledge and the absence of a satisfactory alternative, we consider the establishment of a new genus to be justified.

We hope that the present study will stimulate renewed interest in this species-rich but taxonomically neglected group of predominantly subcortical beetles and encourage future systematic and phylogenetic research that will ultimately resolve the relationships within Platysomatini.

## Supplementary Material

XML Treatment for Aethesoma

XML Treatment for Aethesoma prosternistriatum

## Additional information

### Artificial Intelligence (AI) use

The authors accept full responsibility for the content of the manuscript, including the disclosure of any use of AI.

No AI tools were used in the preparation of this manuscript.

### Author ORCIDs

J. Zheng https://orcid.org/0009-0006-9275-5858

T. Lackner https://orcid.org/0000-0002-0108-5785

### Data availability

All of the data that support the findings of this study are available in the main text.
